# Supplementary material for: Mapping the evolution of fertility support policies in China: A content and instrumental analysis
Source: PLoS One. 2025 Oct 9;20(10):e0332137. doi: 10.1371/journal.pone.0332137 (PMC12510515; doi:10.1371/journal.pone.0332137)
Supplement: S1 Appendix — (ZIP) [file pone.0332137.s001.zip › S1 Appendix. 226 original policy documents/50-国务院办公厅关于促进3岁以下婴幼儿照护服务发展的指导意见(FBM-CLI-2-331919).doc]

国务院办公厅关于促进3岁以下婴幼儿照护服务发展的指导意见

**发布部门：** [国务院办公厅](https://www.pkulaw.com/law/chl?Aggs.IssueDepartment=601) [机构沿革](javascript:void(0))

**发文字号：**国办发〔2019〕15号

**发布日期：**2019.04.17

**实施日期：**2019.04.17

**时效性：** [现行有效](https://www.pkulaw.com/law/chl?Aggs.TimelinessDic=01)

**效力级别：** [国务院规范性文件](https://www.pkulaw.com/law/chl?Aggs.EffectivenessDic=XC0203)

**法规类别：** [妇幼卫生](https://www.pkulaw.com/law/chl?Aggs.Category=09622) [老少妇幼残保护](https://www.pkulaw.com/law/chl?Aggs.Category=01604)

国务院办公厅关于促进3岁以下婴幼儿照护服务发展的指导意见
（国办发〔2019〕15号）

各省、自治区、直辖市人民政府，国务院各部委、各直属机构：
　　3岁以下婴幼儿（以下简称婴幼儿）照护服务是生命全周期服务管理的重要内容，事关婴幼儿健康成长，事关千家万户。为促进婴幼儿照护服务发展，经国务院同意，现提出如下意见。
　　一、总体要求
　　（一）指导思想。以习近平新时代中国特色社会主义思想为指导，全面贯彻党的十九大和十九届二中、三中全会精神，按照统筹推进“五位一体”总体布局和协调推进“四个全面”战略布局要求，坚持以人民为中心的发展思想，以需求和问题为导向，推进供给侧结构性改革，建立完善促进婴幼儿照护服务发展的政策法规体系、标准规范体系和服务供给体系，充分调动社会力量的积极性，多种形式开展婴幼儿照护服务，逐步满足人民群众对婴幼儿照护服务的需求，促进婴幼儿健康成长、广大家庭和谐幸福、经济社会持续发展。
　　（二）基本原则。
　　家庭为主，托育补充。人的社会化进程始于家庭，儿童监护抚养是父母的法定责任和义务，家庭对婴幼儿照护负主体责任。发展婴幼儿照护服务的重点是为家庭提供科学养育指导，并对确有照护困难的家庭或婴幼儿提供必要的服务。
　　政策引导，普惠优先。将婴幼儿照护服务纳入经济社会发展规划，加快完善相关政策，强化政策引导和统筹引领，充分调动社会力量积极性，大力推动婴幼儿照护服务发展，优先支持普惠性婴幼儿照护服务机构。
　　安全健康，科学规范。按照儿童优先的原则，最大限度地保护婴幼儿，确保婴幼儿的安全和健康。遵循婴幼儿成长特点和规律，促进婴幼儿在身体发育、动作、语言、认知、情感与社会性等方面的全面发展。
　　属地管理，分类指导。在地方政府领导下，从实际出发，综合考虑城乡、区域发展特点，根据经济社会发展水平、工作基础和群众需求，有针对性地开展婴幼儿照护服务。
　　（三）发展目标。到2020年，婴幼儿照护服务的政策法规体系和标准规范体系初步建立，建成一批具有示范效应的婴幼儿照护服务机构，婴幼儿照护服务水平有所提升，人民群众的婴幼儿照护服务需求得到初步满足。
　　到2025年，婴幼儿照护服务的政策法规体系和标准规范体系基本健全，多元化、多样化、覆盖城乡的婴幼儿照护服务体系基本形成，婴幼儿照护服务水平明显提升，人民群众的婴幼儿照护服务需求得到进一步满足。

　　二、主要任务
　　（一）加强对家庭婴幼儿照护的支持和指导。
　　全面落实产假政策，鼓励用人单位采取灵活安排工作时间等积极措施，为婴幼儿照护创造便利条件。
　　支持脱产照护婴幼儿的父母重返工作岗位，并为其提供信息服务、就业指导和职业技能培训。
　　加强对家庭的婴幼儿早期发展指导，通过入户指导、亲子活动、家长课堂等方式，利用互联网等信息化手段，为家长及婴幼儿照护者提供婴幼儿早期发展指导服务，增强家庭的科学育儿能力。
　　切实做好基本公共卫生服务、妇幼保健服务工作，为婴幼儿家庭开展新生儿访视、膳食营养、生长发育、预防接种、安全防护、疾病防控等服务。
　　（二）加大对社区婴幼儿照护服务的支持力度。
　　地方各级政府要按照标准和规范在新建居住区规划、建设与常住人口规模相适应的婴幼儿照护服务设施及配套安全设施，并与住宅同步验收、同步交付使用；老城区和已建成居住区无婴幼儿照护服务设施的，要限期通过购置、置换、租赁等方式建设。有关标准和规范由住房城乡建设部于2019年8月底前制定。鼓励通过市场化方式，采取公办民营、民办公助等多种方式，在就业人群密集的产业聚集区域和用人单位完善婴幼儿照护服务设施。
　　鼓励地方各级政府采取政府补贴、行业引导和动员社会力量参与等方式，在加快推进老旧居住小区设施改造过程中，通过做好公共活动区域的设施和部位改造，为婴幼儿照护创造安全、适宜的环境和条件。
　　各地要根据实际，在农村社区综合服务设施建设中，统筹考虑婴幼儿照护服务设施建设。
　　发挥城乡社区公共服务设施的婴幼儿照护服务功能，加强社区婴幼儿照护服务设施与社区服务中心（站）及社区卫生、文化、体育等设施的功能衔接，发挥综合效益。支持和引导社会力量依托社区提供婴幼儿照护服务。发挥网格化服务管理作用，大力推动资源、服务、管理下沉到社区，使基层各类机构、组织在服务保障婴幼儿照护等群众需求上有更大作为。
　　加大对农村和贫困地区婴幼儿照护服务的支持，推广婴幼儿早期发展项目。
　　（三）规范发展多种形式的婴幼儿照护服务机构。
　　举办非营利性婴幼儿照护服务机构的，在婴幼儿照护服务机构所在地的县级以上机构编制部门或民政部门注册登记；举办营利性婴幼儿照护服务机构的，在婴幼儿照护服务机构所在地的县级以上市场监管部门注册登记。婴幼儿照护服务机构经核准登记后，应当及时向当地卫生健康部门备案。登记机关应当及时将有关机构登记信息推送至卫生健康部门。
　　地方各级政府要将需要独立占地的婴幼儿照护服务设施和场地建设布局纳入相关规划，新建、扩建、改建一批婴幼儿照护服务机构和设施。城镇婴幼儿照护服务机构建设要充分考虑进城务工人员随迁婴幼儿的照护服务需求。
　　支持用人单位以单独或联合相关单位共同举办的方式，在工作场所为职工提供福利性婴幼儿照护服务，有条件的可向附近居民开放。鼓励支持有条件的幼儿园开设托班，招收2至3岁的幼儿。
　　各类婴幼儿照护服务机构可根据家庭的实际需求，提供全日托、半日托、计时托、临时托等多样化的婴幼儿照护服务；随着经济社会发展和人民消费水平提升，提供多层次的婴幼儿照护服务。
　　落实各类婴幼儿照护服务机构的安全管理主体责任，建立健全各类婴幼儿照护服务机构安全管理制度，配备相应的安全设施、器材及安保人员。依法加强安全监管，督促各类婴幼儿照护服务机构落实安全责任，严防安全事故发生。
　　加强婴幼儿照护服务机构的卫生保健工作。认真贯彻保育为主、保教结合的工作方针，为婴幼儿创造良好的生活环境，预防控制传染病，降低常见病的发病率，保障婴幼儿的身心健康。各级妇幼保健机构、疾病预防控制机构、卫生监督机构要按照职责加强对婴幼儿照护服务机构卫生保健工作的业务指导、咨询服务和监督检查。
　　加强婴幼儿照护服务专业化、规范化建设，遵循婴幼儿发展规律，建立健全婴幼儿照护服务的标准规范体系。各类婴幼儿照护服务机构开展婴幼儿照护服务必须符合国家和地方相关标准和规范，并对婴幼儿的安全和健康负主体责任。运用互联网等信息化手段对婴幼儿照护服务机构的服务过程加强监管，让广大家长放心。建立健全婴幼儿照护服务机构备案登记制度、信息公示制度和质量评估制度，对婴幼儿照护服务机构实施动态管理。依法逐步实行工作人员职业资格准入制度，对虐童等行为零容忍，对相关个人和直接管理人员实行终身禁入。婴幼儿照护服务机构设置标准和管理规范由国家卫生健康委制定，各地据此做好婴幼儿照护服务机构核准登记工作。

　　三、保障措施
　　（一）加强政策支持。充分发挥市场在资源配置中的决定性作用，梳理社会力量进入的堵点和难点，采取多种方式鼓励和支持社会力量举办婴幼儿照护服务机构。鼓励地方政府通过采取提供场地、减免租金等政策措施，加大对社会力量开展婴幼儿照护服务、用人单位内设婴幼儿照护服务机构的支持力度。鼓励地方政府探索试行与婴幼儿照护服务配套衔接的育儿假、产休假。创新服务管理方式，提升服务效能水平，为开展婴幼儿照护服务创造有利条件、提供便捷服务。
　　（二）加强用地保障。将婴幼儿照护服务机构和设施建设用地纳入土地利用总体规划、城乡规划和年度用地计划并优先予以保障，农用地转用指标、新增用地指标分配要适当向婴幼儿照护服务机构和设施建设用地倾斜。鼓励利用低效土地或闲置土地建设婴幼儿照护服务机构和设施。对婴幼儿照护服务设施和非营利性婴幼儿照护服务机构建设用地，符合《划拨用地目录》的，可采取划拨方式予以保障。
　　（三）加强队伍建设。高等院校和职业院校（含技工院校）要根据需求开设婴幼儿照护相关专业，合理确定招生规模、课程设置和教学内容，将安全照护等知识和能力纳入教学内容，加快培养婴幼儿照护相关专业人才。将婴幼儿照护服务人员作为急需紧缺人员纳入培训规划，切实加强婴幼儿照护服务相关法律法规培训，增强从业人员法治意识；大力开展职业道德和安全教育、职业技能培训，提高婴幼儿照护服务能力和水平。依法保障从业人员合法权益，建设一支品德高尚、富有爱心、敬业奉献、素质优良的婴幼儿照护服务队伍。
　　（四）加强信息支撑。充分利用互联网、大数据、物联网、人工智能等技术，结合婴幼儿照护服务实际，研发应用婴幼儿照护服务信息管理系统，实现线上线下结合，在优化服务、加强管理、统计监测等方面发挥积极作用。
　　（五）加强社会支持。加快推进公共场所无障碍设施和母婴设施的建设和改造，开辟服务绿色通道，为婴幼儿出行、哺乳等提供便利条件，营造婴幼儿照护友好的社会环境。企业利用新技术、新工艺、新材料和新装备开发与婴幼儿照护相关的产品必须经过严格的安全评估和风险监测，切实保障安全性。

　　四、组织实施
　　（一）强化组织领导。各级政府要提高对发展婴幼儿照护服务的认识，将婴幼儿照护服务纳入经济社会发展相关规划和目标责任考核，发挥引导作用，制定切实管用的政策措施，促进婴幼儿照护服务规范发展。
　　（二）强化部门协同。婴幼儿照护服务发展工作由卫生健康部门牵头，发展改革、教育、公安、民政、财政、人力资源社会保障、自然资源、住房城乡建设、应急管理、税务、市场监管等部门要按照各自职责，加强对婴幼儿照护服务的指导、监督和管理。积极发挥工会、共青团、妇联、计划生育协会、宋庆龄基金会等群团组织和行业组织的作用，加强社会监督，强化行业自律，大力推动婴幼儿照护服务的健康发展。
　　（三）强化监督管理。加强对婴幼儿照护服务的监督管理，建立健全业务指导、督促检查、考核奖惩、安全保障和责任追究制度，确保各项政策措施、规章制度落实到位。按照属地管理和分工负责的原则，地方政府对婴幼儿照护服务的规范发展和安全监管负主要责任，制定婴幼儿照护服务的规范细则，各相关部门按照各自职责负监管责任。对履行职责不到位、发生安全事故的，要严格按照有关法律法规追究相关人员的责任。
　　（四）强化示范引领。在全国开展婴幼儿照护服务示范活动，建设一批示范单位，充分发挥示范引领、带动辐射作用，不断提高婴幼儿照护服务整体水平。
　　附件：促进3岁以下婴幼儿照护服务发展工作部门职责分工

国务院办公厅
2019年4月17日

　　附件

　　促进3岁以下婴幼儿照护服务发展工作部门职责分工

　　发展改革部门负责将婴幼儿照护服务纳入经济社会发展相关规划。
　　教育部门负责各类婴幼儿照护服务人才培养。
　　公安部门负责监督指导各类婴幼儿照护服务机构开展安全防范。
　　民政部门负责非营利性婴幼儿照护服务机构法人的注册登记，推动有条件的地方将婴幼儿照护服务纳入城乡社区服务范围。
　　财政部门负责利用现有资金和政策渠道，对婴幼儿照护服务行业发展予以支持。
　　人力资源社会保障部门负责对婴幼儿照护服务从业人员开展职业技能培训，按规定予以职业资格认定，依法保障从业人员各项劳动保障权益。
　　自然资源部门负责优先保障婴幼儿照护服务机构和设施建设的土地供应，完善相关规划规范和标准。
　　住房城乡建设部门负责规划建设婴幼儿照护服务机构和设施，完善相关工程建设规范和标准。
　　卫生健康部门负责组织制定婴幼儿照护服务的政策规范，协调相关部门做好对婴幼儿照护服务机构的监督管理，负责婴幼儿照护卫生保健和婴幼儿早期发展的业务指导。
　　应急管理部门负责依法开展各类婴幼儿照护服务场所的消防监督检查工作。
　　税务部门负责贯彻落实有关支持婴幼儿照护服务发展的税收优惠政策。
　　市场监管部门负责营利性婴幼儿照护服务机构法人的注册登记，对各类婴幼儿照护服务机构的饮食用药安全进行监管。
　　工会组织负责推动用人单位为职工提供福利性婴幼儿照护服务。
　　共青团组织负责针对青年开展婴幼儿照护相关的宣传教育。
　　妇联组织负责参与为家庭提供科学育儿指导服务。
　　计划生育协会负责参与婴幼儿照护服务的宣传教育和社会监督。
　　宋庆龄基金会负责利用公益机构优势，多渠道、多形式参与婴幼儿照护服务。

© 北大法宝：（[www.pkulaw.com](https://www.pkulaw.com/)) 专业提供法律信息、法学知识和法律软件领域各类解决方案。北大法宝为您提供丰富的参考资料，正式引用法规条文时请与标准文本核对。
欢迎查看所有[产品和服务](https://www.pkulaw.com/helps/2.html)。法宝快讯：[如何快速找到您需要的检索结果？](https://www.pkulaw.com/helps/70.html)[法宝](https://www.pkulaw.com/helps/69.html)V6有何新特色？

扫描二维码阅读原文

**原文链接：**<https://www.pkulaw.com/chl/434b24b8b5769383bdfb.html>
